# Diagnostic Potential of Neural Exosome Cargo as Biomarkers for Acute Brain Injury

**DOI:** 10.1002/acn3.499

**Published:** 2017-11-24

**Authors:** Laura Goetzl, Nana Merabova, Nune Darbinian, Diana Martirosyan, Erica Poletto, Keri Fugarolas, Ogechukwu Menkiti

**Affiliations:** ^1^ Departments of Obstetrics & Gynecology Lewis Katz School of Medicine at Temple University Philadelphia Pennsylvania; ^2^ Shriner's Hospital Pediatric Research Center for Neural Repair and Rehabilitation Philadelphia Pennsylvania; ^3^ Department of Radiology Drexel University School of Medicine St. Christopher's Hospital for Children Philadelphia Pennsylvania; ^4^ Departments of Neonatology Drexel University School of Medicine St. Christopher's Hospital for Children Philadelphia Pennsylvania

## Abstract

**Objective:**

Neuronal exosomes purified from peripheral blood samples have been proposed as diagnostic tool in the setting of acute brain injury but never tested clinically. We hypothesized that exosome protein biomarkers would change over time following acute hypoxic brain injury and would predict response to therapy.

**Methods:**

Synaptopodin (SYNPO), an actin‐associated protein present in postsynaptic spines, was evaluated as a potential biomarker as well as: synaptophysin, neuron‐specific enolase, and mitochondrial cytochrome c oxidase. A secondary analysis was performed on neonatal samples collected at 8, 10, and 14 h after the initiation of therapeutic‐controlled hypothermia for acute hypoxic–ischemic encephalopathy (*n* = 14). Neuronal exosomes were purified from serum and protein levels were quantified using standard ELISA methods. The primary study outcomes were length of stay (LOS), discharge on seizure medication (DCMED), and composite neuroimaging score (NIS).

**Results:**

The slope of change in neuronal exosome SYNPO between 8 and 14 h appeared to be the most promising biomarker for all three clinical study outcomes. SYNPO was highly correlated with LOS (−0.91, *P* < 0.001). SYNPO increased in 6/8 without DCMED and was worse or neutral in 5/5 with DCMED (*P* = 0.02). All four neonates with an abnormal NIS had neutral or decreasing SYNPO (*P* = 0.055). Other candidate biomarkers were not associated with outcomes.

**Interpretation:**

This report provides the first clinical evidence that neural exosomes turn over rapidly enough in the peripheral circulation to be used as a “troponin‐like” test following acute brain injury. Optimal sampling and biomarkers likely vary with type of brain injury.

## Introduction

Strategies to decrease adverse neurologic outcomes following acute brain injury require accurate initial diagnosis as well as efficient identification of patients with a suboptimal response to primary treatment to guide early introduction of adjuvant therapies. A common thread linking these two needs is the need for early, rapid, and accurate predictors of degree of acute brain injury and response to therapy. The search for a troponin‐like blood‐based biomarker for the diagnosis of stroke^1^ and traumatic brain injury^2^ has been ongoing but unsatisfying. The biomarkers and biomarker panels that have been evaluated are too numerous to outline here. Many of the difficulties are due to biologic restrictions regarding which markers are able to cross the blood–brain barrier as well as the lack of specificity for neurologic disease over nonspecific acute phase responses. Recently, there has been interest in brain‐derived exosomes that cross the blood–brain barrier, resulting in peripheral blood‐based “liquid brain biopsies”^3^ in contrast with studies that have examined total peripheral exosomes.^4^, ^5^, ^6^ Neural exosome protein cargo‐based diagnostics are promising blood biomarkers of chronic neurologic diseases such as Alzheimer's^7^, ^8^, ^9^, ^10^, ^11^, ^12^ and Parkinson's diseases.^13^ However, to our knowledge, there have been no clinical studies in humans to evaluate the potential of brain‐derived exosomes isolated from peripheral blood in the diagnosis and characterization of acute brain injury.

We have developed a novel technique for purifying fetal neural exosomes from the maternal bloodstream to identify in utero neurologic injury.^14^ We hypothesized that similar techniques could be used to isolate neural exosomes (NEs) from the neonatal peripheral bloodstream and that exosomes biomarkers would correlate with brain injury secondary to acute hypoxia (HIE). An important secondary hypothesis was that NEs would turn over rapidly enough in the peripheral bloodstream that changes in biomarker levels over time that would correlate with therapeutic response to controlled hypothermia, the current standard‐of‐care treatment for neonates with moderate‐to‐severe perinatal hypoxic–ischemic encephalopathy.^15^ Most importantly, we sought to provide a general proof of concept that the cargos of neuronal exosomes purified from blood samples have utility as noninvasive biomarkers of acute brain injury.

## Methods

### Clinical subjects

A secondary analysis was performed on neonatal samples collected as part of a prospective, open‐label pharmacokinetic study conducted at St. Christopher's Hospital for Children (Philadelphia, PA) to study the pharmacokinetics of ampicillin in a prospective cohort of term neonates undergoing therapeutic‐controlled hypothermia for HIE^16^ The study protocol was approved by the Drexel University College of Medicine Institutional Review Board. Eligibility criteria for inclusion were a gestational age (GA) >36 weeks, birthweight (BW) >1.8 kg, age < 6 h at the time of admission to the NICU, cord gas or arterial or venous blood gas with a pH < 7.0 or a base deficit >16 mEq/L of sodium bicarbonate within the first hour of life, or the presence of seizures and/or evidence of moderate‐to‐severe encephalopathy (apnea, lethargy, hypotonia, or hypertonia). Neonates without central venous access established by 12 h of life were excluded from this investigation to prevent pain or discomfort associated with blood sampling. Following informed consent, 0.5 mL of blood was collected at 8, 10, and 14 h after the initiation of hypothermia coinciding with other scheduled laboratory investigations. Residual aliquots from serum used to assess ampicillin levels were stored at −70°C for a period of 2–3 years.

### Isolation of neonatal neural exosomes

**Figure 1:**
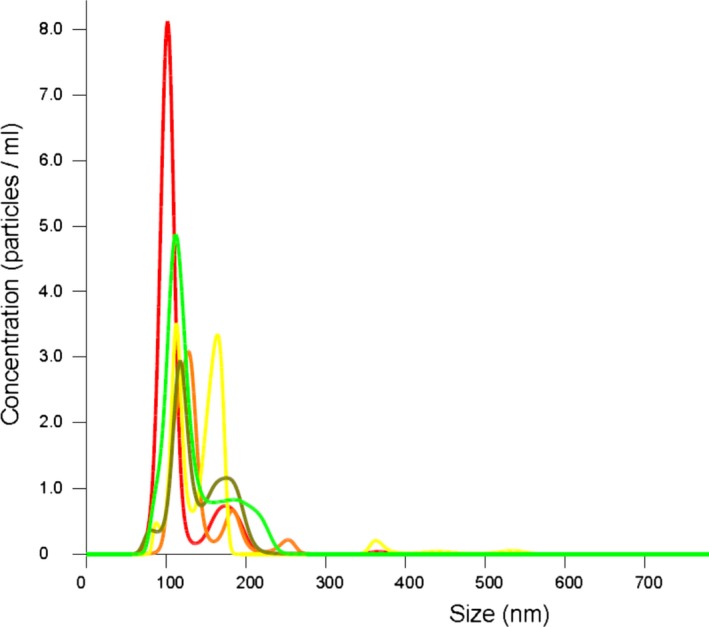
Nanoparticle‐tracking analysis of NNEs. Nanoparticle‐tracking analysis revealed a mean particle diameter of 134 nm ± 46.6 nm and a mode is 109.8 nm

Neonatal neuronal exosomes were purified from residual serum aliquots using previously described techniques.^14^ Briefly, 100 *μ*L of serum was incubated with thromboplastin‐D and a cocktail of protease and phosphatase inhibitors. After centrifugation, supernates were incubated with exosome precipitation solution (EXOQ; System Biosciences, Inc., Mountainview, CA). To isolate the subset of exosomes from neural sources, total exosome suspensions were incubated with monoclonal IgG1 anti‐human Contactin‐2/TAG1 antibody (clone 372913, R&D Systems, Inc., Minneapolis, MN) that had been biotinylated (EZ‐Link sulfo‐NHS‐biotin System, Thermo Scientific, Inc.), and antibody‐bound exosomes were precipitated with Streptavidin‐Plus UltraLink Resin (Pierce‐Thermo Scientific, Inc.). Contactin‐2/TAG1 is a glycosylphosphatidylinositol‐anchored neuronal membrane adhesion protein of the immunoglobulin superfamily that is transiently expressed in human brain development to guide axonal connections and, in association with other proteins, promote molecular organization of myelinated nerves.^17^, ^18^ Nanoparticle‐tracking analysis was performed which revealed a mean diameter of 134 nm ± 46.6 nm and a mode is 109.8 nm (Fig. 1). This is consistent with exosomes (generally 50‐150 nm). Minor contamination with microvesicles (generally 200–1000 nm) cannot be excluded.

### Quantification of protein biomarkers

Potential protein biomarkers including synaptopodin (SYNPO), synaptophysin (SYN), neuron‐specific enolase (NSE) and mitochondrial cytochrome c oxidase (COX IV) were quantified using standard ELISA methods. The slope of biomarker levels between 8 h and 14 h was calculated to assess whether or not marker levels were rising or falling over the course of therapy. Slopes (ng/mL/hr) were utilized as we were concerned that individual values at a single time point might vary significantly with the wide ranges of gestational age and birthweight. Slopes calibrated values for each infant against their starting value and eliminated the need for repeated measures analysis. If an 8‐h sample was not available, the slope between 10 h and 14 h was substituted (total of two occurrences). One subject had only one sample, and slope could not be calculated.

### Neuroimaging

A neuroradiologist (EP) scored available T1/T2 and DWI magnetic resonance images from between day of life 5 and 7 in a blinded fashion according to the scoring system described by Barkovich that correlates with neuromotor outcome at ages 3 and 12 months.^19^ T1‐weighted, T2‐weighted, and diffusion‐weighted images were analyzed for signal abnormality within the basal ganglia (BG) or watershed (W). Basal ganglia abnormality scores range from 0 to 4, with 4 being the most extensive abnormality. Watershed abnormality scores range from 0 to 5, with 5 being the most extensive abnormality. A summary score (NIS) was calculated based on the arithmetic sum of the basal ganglia and watershed scores from T1/T2‐weighted and DWI images.

### Data analysis

The primary study outcomes, selected prior to data analysis, were length of stay (LOS, continuous), discharge on seizure medication (DCMED, categorical binary), and NIS (Ordinal, individual and summary scores). Long‐term neurologic outcomes were not uniformly available for this cohort. Data analyses were performed using IBM SPSS Statistics for Windows (Version 24.0. Armonk, NY: IBM Corp). The small sample size did not allow for adequate assessment of the normality of the data, and therefore, nonparametric statistical methods were chosen. Medians were compared using the independent samples median test. Ordinal categories are compared using Somers' d test. Bivariate correlations were assessed by Spearman's rho. A *P* < 0.05 was considered statistically significant.

## Results

The clinical characteristics of the 14 neonates enrolled are shown (Table 1). Consistent with the criteria for controlled hypothermia, first pH, base excess (BE), and 5‐min APGAR score were significantly abnormal. The majority of neonates were male, limiting our ability to stratify results by neonatal sex. Only one infant was exposed to chorioamnionitis. The majority of encephalopathy was grade 2; therefore, we could not stratify results by Sarnat score. About 50% of cases were associated with an acute obstetric event such as abruption, uterine rupture, or cord prolapse. Thirteen neonates had T1/T2‐weighted images available for analysis; 12 neonates had DWI images.

**Table 1:** Clinical characteristics of the cohort

Subject	Gestational age	Birthweight	Sex	APGAR 5 Min	pH	BE	Sarnat	Chorio	OB event
A	37.1	2945	M	4	7.0	−23	2	No	Yes
B	41.3	3737	M	9	7.0	−20	2	No	No
C	39.6	3440	M	0	6.7	−32	3	No	Yes
D	38.0	2975	M	0	7.1	−17	2	No	Yes
E	40.1	2415	M	5	7.2	−11	2	No	No
F	40.7	3170	M	2	7.2	−16	2	No	No
G	40.7	4850	M	2	7.0		2	No	Yes
H	40.0	3030	F	0	6.7	−34	3	No	Yes
I	39.3	3218	M	1	6.9	−15	2	No	No
J	36.0	2710	F	1	6.7	−28	2	No	Yes
K	36.7	3215	M	2	7.0	−16	2	Yes	No
L	39.0	3760	F	0	6.7	−29	2	No	No
M	38.7	3165	F	2	6.9	−25	2	No	Yes
**N**	41.0	3757	M	2	7.0	−22	2	No	Yes

Chorio, chorioamnionitis, BE, base excess, OB event, acute obstetric event.

**Figure 2:**
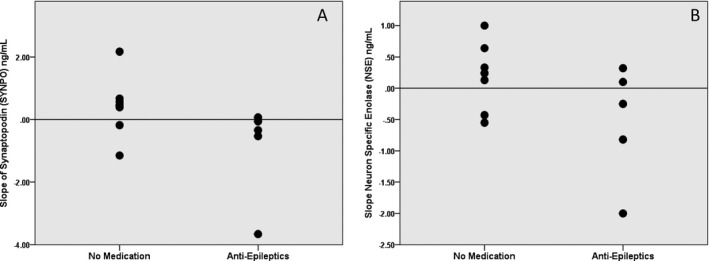
Discharge on seizure medications. The slope of neural exosome and biomarkers levels between 8 and 14 h of controlled hypothermia or initial clinical biomarkers in patients requiring discharge on anticonvulsants compared to patients able to be discharged without medication. (A) Synaptopodin (SYNPO,* P* = 0.02), (B) neuronal‐specific enolase (NSE,* P* = 0.27)

The correlation between the slopes of the four biomarkers and LOS was assessed (Table 2). The slopes of NNE SYN and NSE were significantly positively correlated; however, there was no other relationship between any other biomarker pair. The slope of NNE SYNPO was highly negatively correlated with LOS; decreasing NNE SYNPO over time was associated with longer LOS (*r* = −0.91, *P* < 0.001). No other biomarker slope was associated with LOS. As expected, of the traditional clinical indicators (BE, pH, APGAR score), only pH was weakly correlated with LOS (Table 3). Median biomarker slopes were compared between neonates requiring discharge on anticonvulsants compared to neonates able to be discharged without medication (Fig. 2A and B). Median NNE SYNPO slope was significantly lower in neonates requiring anticonvulsants (*P* = 0.02). Perhaps more telling, no infant discharged on anticonvulsants had an increasing NNE SYNPO slope; all slopes in this category were neutral or decreasing. Improving levels of neuronal exosome synaptopodin were exclusively associated with medication‐free discharge. Median NSE slope was not different between discharge outcome categories. Similarly, NNE SYN and COX IV showed no specific pattern (Cox IV, *P* = 1.0; SYP, *P* = 0.59, data not shown). Again as expected, there was sufficient overlap between positive and negative outcomes with traditional predictors such as pH, base excess, and APGAR scores to render them difficult to use for counseling or to make additional treatment decisions after the initial decision for the induction of controlled hypothermia (data not shown).

**Table 2:** Correlations between exosome biomarker slope between 8 and 14 h of head cooling and with length of stay

	SYNPO	NSE	COX IV	LOS
SYN	*r* = 0.17 *P* = 0.58	*r* = 0.75 *P* = 0.003	*r* = 0.23 *P* = 0.45	*r* = −0.15 *P* = 0.63
SYNPO		*r* = 0.22 *P* = 0.48	*r* = −0.30 *P* = 0.32	*r* = −0.91 *P* < 0.001
NSE			*r* = 0.26 *P* = 0.38	*r* = −0.14 *P* = 0.65
COX IV				*r* = 0.26 *P* = 0.40

SYN, synaptophysin; SYNPO, synaptopodin; NSE, neuron‐specific enolase, COX IV, mitochondrial cytochrome c oxidase, LOS, length of stay.

**Table 3:** Correlations between clinical predictors and length of stay

	APGAR 5 min	Base excess	pH	LOS
APGAR 5 min		*r* = 0.63 *P* = 0.02	*r* = 0.55 *P* = 0.04	*r* = −0.31 *P* = 0.29
Base excess			*r* = 0.76 *P* = 0.002	*r* = −0.35 *P* = 0.24
pH				*r* = −0.54 *P* = 0.05

**Figure 3:**
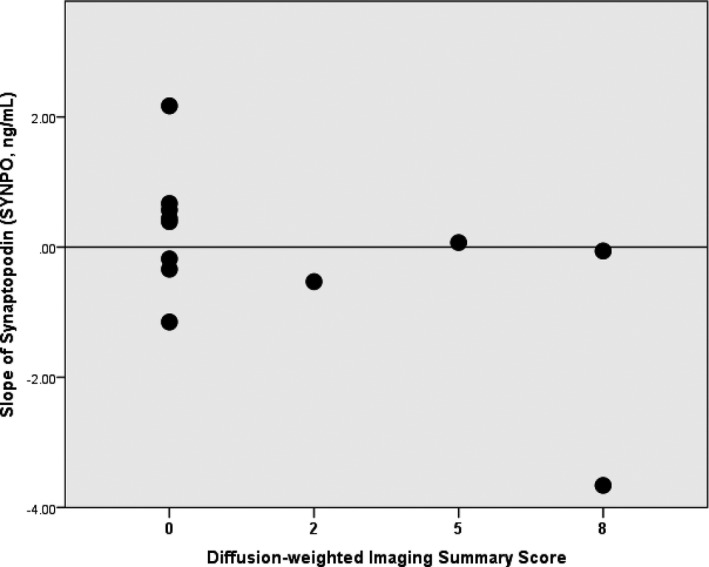
Synaptopodin and Diffusion‐weighted Imaging Summary Score. The slope of neural exosome synaptopodin (SYNPO) levels between 8 and 14 h of controlled hypothermia and arithmetic sum of score from diffusion‐weighted images in basal ganglia and watershed (DS) (*P* = 0.055). Data not shown T1/T2 Summary Score, *P* = 0.15

No strong correlations were identified between synaptopodin slope and any individual scores or with the T1/T2‐weighted summary measure (data not shown, *P* = 0.15). However, we saw a statistically significant relationship between the summary score for the diffusion‐weighted images and the slope of synaptopodin (Fig. 3). All patients with an elevated DWI summary score had a neutral or worsening slope for NNE SYNPO (*P* = 0.055). The worst NNE SYNPO slope (−3.66) was seen in a subject with imaging results consistent with an intraventricular hemorrhage and early‐onset hydrocephalus. One subject with a deteriorating NNE SYNPO slope was not included as that neonate did not undergo DWI.

## Discussion

We present novel data that confirm our two initial hypotheses. First, neural exosomes can be isolated from peripheral blood samples and contain biomarkers that are associated with clinically significant short‐term neurologic outcomes. Second, the turnover of neural exosomes in the peripheral blood compartment is rapid enough that changes in exosome cargo can be assessed at clinically useful intervals to estimate therapeutic response. This allows for early discrimination between responders and nonresponders, facilitating timely adoption of additional measures where available. Finally, we were able to demonstrate that exosome‐based tests can be performed on very small clinical samples that are reasonable to collect serially from critically ill subjects (100 *μ*L). Taken together, this evidence suggests that neural exosome biomarkers are a potentially powerful clinical tool in the classification of the severity of neonatal HIE and the response to therapy. However, the implications of this report are broader than neonatal HIE as they suggest that neuronal exosomes may have diagnostic utility in other forms of acute brain injury such as stroke or trauma.

Exosomes are cell‐derived vesicles that consist of cytoplasm packaged in a membrane layer.^20^ Originally thought to be a cellular mechanism for disposal, exosomes are now known to have important functions including immune regulation, modulation of inflammation, cell‐to‐cell signaling, and angiogenesis. Exosomes are known to be released from living neurons as part of normal neuronal development as important drivers of synaptic plasticity.^21^, ^22^, ^23^ Increased levels of exosomes and microparticles have been reported to be released from cells after traumatic brain injury^24^ and stroke,^25^ which may increase peripheral exosome yields. Some of the characteristics of exosomes that increase their value as biomarkers include the following: (1) Exosomes carry surface transmembrane proteins that can be representative of their cell of origin and allow identification and purification, (2) the surface of exosomes is known to contain targeting and adhesion molecules that may be important in synaptogenesis and neuronal migration and represents important potential markers of abnormal neurodevelopment, and (3) unlike free protein and miRNA, exosomal cargo is protected from degradation in the circulation, enhancing biomarker recovery. Neuroexosome protein biomarkers retain useful predictive utility despite freezer storage of up to 10 years.^7^, ^8^

Synaptopodin emerged as the most promising biomarker in this investigation. Synaptopodin is a cytoskeletal protein that is essential for the formation of dendritic spines in telencephalic neurons, and is a key mediator of synaptic plasticity, especially at excitatory synapses.^26^, ^27^, ^28^, ^29^ Reduced to essentials, synaptopodin binds actin and, through a calcium‐dependent mechanism, helps maintain normal synaptic structure. We hypothesize that damaged neurons scavenge synaptopodin from exosomes to help repair cellular damage; therefore, SYNPO depletion in exosomes is a measure of neuronal injury. Only a few other clinical studies have reported on blood‐based exosome biomarkers in the setting of acute brain injury.^4^, ^5^ Chen et al. reported on the association between circulating exosomal miRNA‐223 and outcomes following acute ischemic stroke.^4^ Ji et al. reported on the association between circulating exosomal miRNAs 9 and 124 in the setting of acute ischemic stroke.^5^ A key difference between these studies and ours is that both tested total peripheral exosomes instead of the subset of neuronally derived exosomes which may have accounted for the relatively weak (although statistically significant) associations they reported. We hypothesize that restricting analyses to the neuronal subset of exosomes will increase the negative and positive predictive values of biomarkers.

Our study has several limitations. The first and most critical was the small amount of residual serum available for exploratory studies. This forced us to be parsimonious with our pilot markers, and we were only able to assess a limited candidate group. It was difficult to choose from among available candidate biomarkers as none have proven particularly clinically useful. In a recent meta‐analysis, serum and CSF concentrations of interleukin‐1b, interleukin‐6, and serum NSE were predictive of abnormal outcomes in HIE.^30^ We did not include inflammatory cytokines as they are more likely to characterize delayed rather than early neuronal injury and death. We chose synaptopodin and synaptophysin based on their utility as biomarkers of fetal brain injury following alcohol exposure (published and unpublished data).^14^ We included COX IV as a marker of mitochondrial oxidative stress, but it was not discriminatory. This is likely due to the low prevalence of mitochondria in exosomes as well as the fact that oxidative stress may be more likely to result in protein phosphorylation rather than a quantitative change. We included NSE as a potential candidate marker of early neuronal injury in the setting of therapeutic hypothermia based on its prior use in this setting.^31^, ^32^, ^33^ However, in retrospect, it is not surprising that NSE did not yield robust results. NSE is likely released from dying neurons as a marker of cell disintegration. In contrast, neural exosomes production may require largely intact intracellular mechanisms.^34^, ^35^, although extracellular vesicles can also be produced during apoptosis via an alternative “beads on a string” mechanism.^36^ A second serious limitation was our incomplete protocol adherence with missing serum samples preventing the calculation of SYNPO slope in one patient and missing DWI occurring in the subject with the worst SYNPO slope (likely because the subject was too ill to undergo imaging). However, despite these further reductions in usable sample size, we were able to generate robust data for synaptopodin and demonstrate a significant association with all three study outcomes. A third limitation was the absence of long‐term neurologic follow‐up in subjects.

Overall, we are confident that the data we present support further prospective studies of neural exosomes as early, noninvasive biomarkers in the setting of acute brain injury. Serial sampling may or may not prove superior to single time point testing; in prospective trials, we suggest that the first sampling point be set to coincide with primary treatment initiation. Additional markers should be investigated in addition to confirmatory studies of synaptopodin that take into account the two phases of neuronal injury^37^: primary energy failure/hypoxia (<6 h after injury) and the secondary energy failure accompanied by oxidative stress, excitotoxic injury, and inflammation (>6 h after injury). Any biomarker tests of traumatic brain injury would be maximally clinically effective with the shortest turnaround time. The full protocol performed in this set of experiments required >24 h; if performed using high‐throughput methods, it could be performed in as few as 4 h. Recent advances suggest that simple paper‐based tests^38^ and smartphone‐based microfluidic‐based mobile exosome detectors^39^ are imminent which will reduce turnaround time to 1 h or less. In summary, neuronal exosome assays provide an early and noninvasive window into brain response to therapy for acute injury. Further research is needed to identify the optimal sampling strategy and to refine the negative and positive predictive values with regard to long‐term outcomes.

## Conflict of Interest

Only one author has a potential conflict of interest. Dr. Goetzl has applied for a patent for some of the methodology described but has no current licensing or industrial applications.
